# New insights on ill-thriftiness in early-weaned buffalo calves

**DOI:** 10.14202/vetworld.2016.579-586

**Published:** 2016-06-12

**Authors:** Nasr-Eldin M. Aref, Ali El-Sebaie, Hammad Zaghloul Hammad

**Affiliations:** 1 Department of Animal Medicine, Faculty of Veterinary Medicine, Assiut University, Assiut, Egypt; 2 Animal Health Research Institute, Sohag, Egypt

**Keywords:** calves, growth, hormone, insulin, insulin-like growth factor-1, weaning

## Abstract

**Aim:**

The present study was designed to: (1) Investigate the effect of weaning time on various metabolic indices and growth pattern in buffalo calves compared to cow calves under field condition and (2) Shed light on the potential relationship between early weaning, growth metabolites, and suboptimal growth (ill-thrift) in buffalo calves.

**Materials and Methods:**

A total number of 18 neonatal calves of both sexes and species (cattle and buffalo) were included in the study. Animals were divided into three groups according to their age at weaning as following: Cow calves (n=8) weaned at 4.5 months, buffalo calves (n=6) weaned at 3.5 months (early-weaned), and buffalo calves (n=4) weaned at 5.5 months (late-weaned). Morphological traits, growth metabolites, and hormonal profile were measured at monthly interval over the period of the study and around the time of weaning (2 weeks pre- and post-weaning).

**Results:**

The obtained results showed that the trend of growth pattern was significantly increased in a linear pattern in cow calves and late-weaned buffalo calves, whereas early-weaned buffalo calves showed sharp decline in their body weight (BW) post-weaning. By the end of the study, early-weaned buffalo calves showed the lowest BW gain (ill-thrift). There is a positive association between the morphological traits and various growth metabolites and hormonal indices. A significant decrease (p<0.05) in the concentrations of growth hormones (insulin-like growth factor-1 [IGF-1] and insulin) and other metabolites were reported in early-weaned buffalo calves compared to other animals. There is no association between stress indices (cortisol level and neutrophil to lymphocyte ratio) and growth rate.

**Conclusion:**

Suboptimal growth rate (ill-thriftiness) is common in early-weaned buffalo calves and is attributed to low blood levels of growth metabolites, in particularly, IGF-1. In addition, the strong positive associations between concentrations of IGF-1 and morphological characters of growth suggest that IGF-1 is a reliable indicator for assessing metabolic status of individual calves.

## Introduction

Livestock production is an important part of the national economy and is an integral component of sustainable food production. However, there are several challenges to achieve an efficient production system and consequently good livestock profitability. One of the most important production challenges for the livestock industry is to maintain a normal growth rate throughout the animal’s life [1].

Growth is a dynamic process which is regulated by several factors including metabolic hormones (growth hormone [GH], thyroid hormones, insulin, and leptin), nutritionally-related metabolites (protein, glucose, lipid, minerals, and vitamins), and growth factors (insulin-like growth factor-1 [IGF-1]) [2]. The changes in circulating concentrations of these metabolites are important signals of the metabolic status of growing animals [3]. The somatotropic axis is the most important hormonal system for growth, which primarily consists of GH, IGF-1, and their carrier proteins and receptors [4,5].

Suboptimal growth or ill-thrift is a common problem for many of livestock producers. While there are several risk factors associated with ill-thrift in calves, the transition period of weaning is considered an important factor for poor growth rate especially for early-weaned animals [6]. Weaning is a multifactorial stress or in which socio-psychologic, nutritional, and physical stressors are combined. Psychological stress is present in the form of maternal separation and social disruption, whereas physical and nutritional stressors are often present in the introduction of/and adaptation to a new diet and a new environment [7].

Comparing to cow calves, buffalo calves may struggle to accomplish a successful weaning transition. Therefore, slaughtering of recently-weaned buffalo calves is a common trend in Egypt to avoid the cost of management and delayed marketing.

Following earlier studies on cow calves, the transitional period of weaned buffalo calves needs an in-depth exploration to elucidate the different factors that contribute to growth retardation. The analysis of growth indicators is a reliable method for the evaluation of growth pattern [8]. In addition, using of growth biomarkers may offer predictive value for suboptimal growth (ill-thrift). Our hypothesis proposed that early-weaned buffalo calves experience different concentrations of growth metabolites than late-weaned buffalo and cow calves.

Therefore, the present study was carried out to: (1) Determine the morphological characters of growth (body weight [BW], height, girth, crown ramp length (CRL), and average daily change [ADC]) in weaned buffalo and cow calves, (2) Evaluate the indices of growth metabolites (glucose, total protein [TP], urea, insulin, and IGF-1) and stress (cortisol, albumin/globulin [A/G] ratio, white blood cells [WBCs], and neutrophil to lymphocyte ratio [N/L] ratio) in weaned buffalo and cow calves, and (3) Determine the relationship between the morphological characters of growth and blood biomarkers of growth metabolites and stress in weaned buffalo and cow calves.

## Materials and Methods

### Ethical approval

The present study was approved by the Institutional Animal Ethics Committee of The Faculty of Veterinary Medicine at Assiut University.

### Animals farm and weaning strategy

A total number of 18 neonatal calves of both sexes and species (cattle and buffalo) were included in the study. Animals were divided into three groups according to their age at-weaning time as following: Cow calves (n=8) weaned at 4.5 months, buffalo calves (n=6) weaned at 3.5 months (early-weaned), and buffalo calves (n=4) weaned at 5.5 months (late-weaned). These animals were belonging to a private commercial feedlot farm located 10 km north to Sohag city-Egypt. Farm adopted traditional weaning method. Briefly, calves present together with their dams suckling whole milk *ad libitum*. No milk replacer provided to calves. At the time of weaning, calves were separated from their dams and moved into a dry lot pen with feed bunks and water troughs. Calves were fed on straw mixture (wheat straw, alfalfa hay, caraway hay, cumin hay, and fenugreek hay) as roughage components and concentrate mixture to meet their nutrient requirement for a daily gain. Concentrate mixture consisted of soybean cake and cottonseed meal (25%), maize crushed (40%), wheat bran (32%), mineral mixture (2%), and common salt (1%). High-quality green fodders offered *ad libitum* to calves with clean drinking water. No changes in the aspects of calf’s management over the period of study.

### Determination of morphological characters of growth

All calves were subjected to assessment of BW and the body morphological measurements including: Heart girth, height at withers, and CRL. Initial BWs and body morphological measurements of the animals were recorded at the start of the study, monthly over the period of the study, and around the time of weaning (2 weeks pre- and post-weaning). BW (BW/Kg) was measured using a portable weight platform; heart girth (G/cm) and CRL (CRL/cm) were measured using a tape measure; height at withers (HT/cm) was measured using a height stick. All characteristics were measured by the same person. The ADC of the morphological characters of growth (weight, height, girth, and CRL) was calculated pre-weaning, post-weaning, and all over the study period for each calf. The total ADC was determined mathematically by subtraction the starting measurements from the end measurement and divided by the number of days between the two time points, to give the total ADC in each morphological characters of growth over the study period. Pre-weaning ADC was calculated subtracting start measurements from the weaning day measurements and divided by the number of days between the two time points to give the ADC in each parameter. Post-weaning ADC was similarly calculated by subtracting weaning day measurements from the end measurements and divided by the number of days between the two time points to give the ADC in each parameter.

### Clinical examination

The health status of the studied animals was monitored through careful clinical examination according to Cockcroft [9].

### Blood sampling, timing, and assays methods

Blood and serum samples were collected according to Otter [10]. Whole blood samples were used for CBC, whereas serum samples were used for determination of TP (g/L), albumin (g/L), urea (mmol/L), insulin (µIU/mL), IGF-1 (ng/mL), and cortisol (ng/mL). Samples were collected at a monthly interval from the start to the end of the study. Additional samples were collected around the times of weaning (2 weeks pre- and 2 weeks post-weaning) at weakly interval to elucidate the impact of weaning on growth and stress parameters.

Total leukocytic count (×10^6^/L), neutrophils (%), and lymphocytes (%) were determined using an automated hematology analyzer (ADVIA 2120, Bayer Healthcare, Siemens, UK) equipped with software for bovine blood. N/L ratio was calculated by dividing the percent of neutrophils by percent of lymphocytes for each sample.

Serum biochemical analysis including TP (g/L), albumin concentration (g/L), serum glucose level (mmol/L), and serum urea (mmol/L) were determined spectrophotometrically using diagnostic test kits (Gesellschaft fϋr Biochemica und Diagnostica GmbH, Germany). Serum globulin level (g/L) was determined mathematically by subtraction of the serum albumin level (g/L) from the serum TP level (g/L). A/G ratio was calculated by dividing the values of serum albumin by serum globulin.

IGF-1 (ng/mL) and cortisol concentrations (ng/mL) were measured by DRG IGF-1 600 enzyme-linked immunosorbent assay (ELISA) kit (EIA-4140) and DRG cortisol ELISA Kit (EIA-1887), respectively (DRG Diagnostics, GmbH, Germany, Division of DRG International, Inc.). Both tests are solid phase ELISA for quantitative *in vitro* diagnostic measurement of IGF-1 and cortisol based on the principle of competitive binding. The intensity of color developed is reverse proportional to the concentration of hormone in the sample. All technical procedures described by the manufacturer were followed.

Estimation of insulin level (µIU/mL) was performed using DRG Insulin ELISA kit (EIA-2935) (DRG Diagnostics, GmbH, Germany, Division of DRG International, Inc.). The DRG Insulin ELISA kit is a solid phase ELISA based on the sandwich principle. The intensity of color developed is proportional to the concentration of insulin in the sample. All technical procedures described by the manufacturer were followed.

A microtiter plate reader (Stat Fax - 2100, Awareness, Technology Inc., USA) provided with printer EPSON-LX 300+ was used to read the ELISA plate.

### Statistical analysis

Data were analyzed using the packaged SPSS program for Windows version 10.0.1 (SPSS Inc., Chicago, IL, USA). Differences between groups were determined by the one-way analysis of variance followed by the Student’s *t*-test or the pairwise multiple comparison procedures (when significant *F*-test was found) using Duncan’s new multiple range test. Data were presented as mean ± standard error (Standard error of the mean). Pearson correlation coefficients (r) were determined between paired variables for individual animals for cow calves (n=8) and buffalo calves (n=10). The significance level was set at p≤0.05.

## Results

### Growth curve

**Figure-1:**
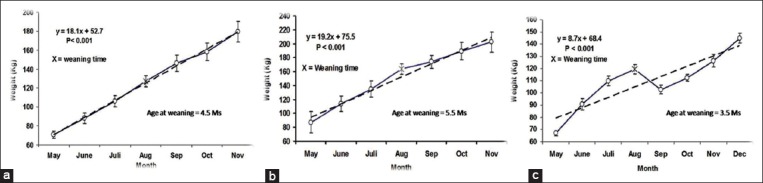
Pattern of growth curve of cow calves (a), late-weaned (b), and early-weaned buffalo calves (c) group.

Time-course evaluation of growth patterns of calves over the studied period is shown in Figure-1. Both cow and late-weaned buffalo calves groups showed a consistent gain of BW as indicated by an increasing linearity of growth curve (Figure-1a and b) while early-weaned buffalo calves experienced a marked drop in the BW post-weaning (Figure-1c) and could not achieve the same BW of the other 2 groups at the end of the study.

### BW (BW/kg)

**Figure-2:**
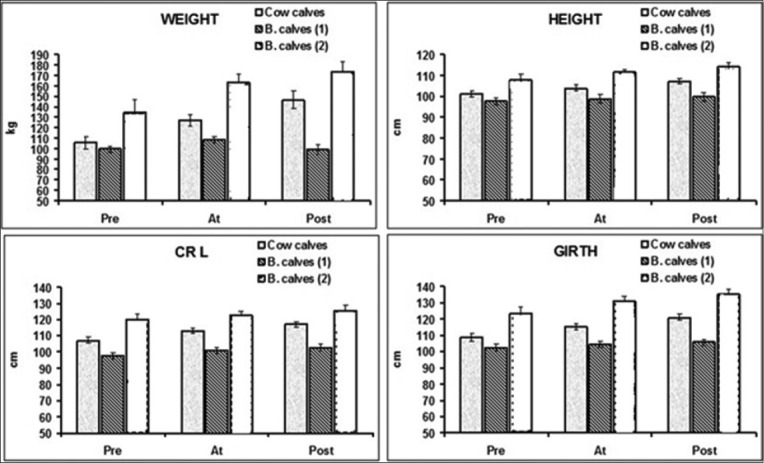
Time-course evaluation (mean±standard error) of morphological traits of cow calves, late- and early-weaned buffalo calves group.

BW (kg) showed significant differences between groups over the period of the study (Figure-2). The prominent feature was a significant decrease in the BW in early-weaned buffalo calves compared with cow and late-weaned buffalo calves. The ADCs of BW (kg/day) at pre-weaning period of cow calves, early-weaned buffalo calves, and late-weaned buffalo calves were 0.65±0.04, 0.75±0.05, and 0.88±0.06 kg/day, respectively, while in the post-weaning period were 0.55±0.06, 0.30±0.1, and 0.48±0.03, respectively. The results showed a significant decrease in ADC of BW in early weaned buffalo calves compared with the other two groups over period of the study. Within the same group, there were no significant differences in the ADC of BW between the pre-weaning and post-weaning periods in cow calves while there was significantly decrease in the ADC of BW in both early- and late-weaned buffalo calves at post-weaning compared with the pre-weaning period.

### Height (cm)

There were significant differences in height (cm) between all groups over the period of the study (Figure-2). At the pre-weaning time, there were significant increases in the height of late-weaned buffalo calves compared with other two groups. Within the same group, all calves showed significantly increase in the heights at post-weaning compared with pre-weaning time. There were significant differences in ADC of height (cm/day) between the three groups over the period of the study. Late-weaned buffalo calves showed significantly higher ADC of height (cm/day) than the other two groups. Within the same group, ADC of height (cm/day) was significantly decreased in all groups at post-weaning compared with the pre-weaning period.

### Girth (cm)

There were significant differences in girth and ADC of girth between all groups of the study (Figure-2). For cow calves, early- and late-weaned buffalo calves, the mean values of girth at pre-weaning were 108.75±2.43, 102.33±2.08, and 123.75±3.54, respectively, whereas at post-weaning were 121.12±1.96, 105.67±1.89, and 135.25±3.09, respectively. Late-weaned buffalo calves showed significant higher girth than other groups over the period of the study. Within the same group, cow calves showed significantly increase girth (cm) post-weaning, whereas other two groups of buffalo calves showed no significant differences in girth. The ADC of girth (cm/day) in cow calves was significantly higher than buffalo calves groups at post-weaning period. Within the same group, the ADC of girth was significantly lower in early-weaned buffalo calves at post-weaning compared with pre-weaning periods.

### CRL (CRL/cm)

CRL (cm) showed significant differences between all groups over the period of the study (Figure-2). Within the same group, CRL (cm) was significantly increase in cow calves at-weaning time and post-weaning period compared to the pre-weaning period of the study. The ADC of CRL (cm/day) in early-weaned buffalo calves was significantly lower compared with the other two groups over the period of the study. Within the same group, post-weaning period the ADC of CRL (cm/day) was significantly lower than pre-weaning times in all groups.

### Clinical observation

Abnormal clinical signs were observed in only two calves of early-weaned group in the form of diarrhea (3-6 days post-weaning) and spontaneously resolved within 2 days without treatment. The animals of this group showed signs of ill-thriftiness in the form of stunting or delayed growth with alopecia, rough hair, poor body condition, emaciation, and pale mucous membrane.

### Metabolic and stress indices

**Figure-3:**
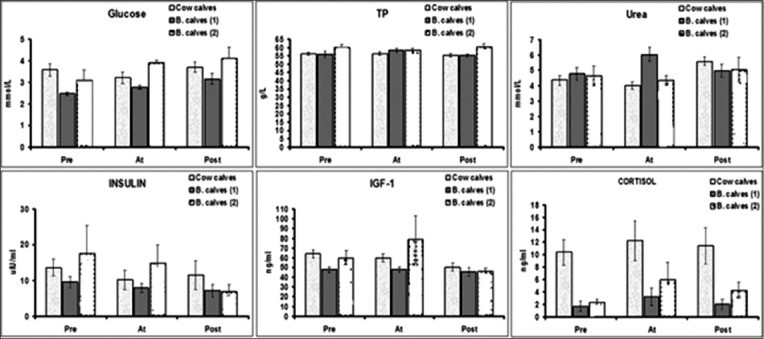
Time-course evaluation (mean±standard error) of biochemical, hormonal, and stress indices of cow calves, late- and early-weaned buffalo calves group.

The analysis of biochemical, hormonal, and stress indices including serum glucose, urea, TP, albumin, insulin, IGF-1, and cortisol were presented in Figure-3.

### Relationships between metabolic variables with the growth rates

The correlation coefficient between the morphological characters of growth and various metabolites of the cow and buffalo calves was presented in Table-1. Obtained data showed strong positive correlations between IGF-1 and all morphological characters of growth. There were also positive correlations between insulin and height and girth. Glucose level was found to be associated with height, girth, and CRL. The urea concentration showed significant relationships with height, girth, and CRL in buffalo calves while it is associated with girth and CRL in cow calves. There was also an association between protein profile and morphological characters of growth. There was no correlation between cortisol level and morphological characters of growth.

**Table-1:** Pearson correlation coefficients (r) between biochemical, hormonal, and physical indices in cow and buffalo calves.

Parameter	Glucose	TP	Albumin	Globulin	Urea	Insulin	IGF-1	Cortisol
Height								
Cow calves								
r	0.331	0.366	0.134	0.355	0.229	0.289	0.598	0.022
P	0.009	0.003	0.298	0.003	0.061	0.019	0.000	0.851
Buffalo calves								
r	0.386	0.425	0.175	0.402	0.301	0.355	0.688	0.031
P	0.008	0.002	0.256	0.002	0.048	0.019	0.000	0.765
Weight								
Cow calves								
r	0.133	0.158	0.101	0.276	0.016	0.189	0.328	0.218
P	0.296	0.199	0.410	0.029	0.865	0.127	0.007	0.081
Buffalo calves								
r	0.186	0.213	0.154	0.344	0.022	0.256	0.394	0.264
P	0.205	0.198	0.372	0.021	0.788	0.102	0.006	0.079
Girth								
Cow calves								
r	0.401	0.458	0.278	0.375	0.318	0.426	0.579	0.109
P	0.002	0.000	0.018	0.003	0.013	0.001	0.000	0.364
Buffalo calves								
r	0.453	0.499	0.352	0.452	0.354	0.468	0.644	0.156
P	0.001	0.000	0.012	0.002	0.009	0.000	0.000	0.325
CRL								
Cow calves								
r	0.627	0.276	0.187	0.207	0.264	0.204	0.428	0.138
P	0.000	0.024	0.121	0.097	0.044	0.113	0.001	0.246
Buffalo calves								
r	0.686	0.344	0.251	0.246	0.302	0.255	0.493	0.170
P	0.246	0.024	0.121	0.097	0.044	0.113	0.001	0.000

Correlation is significant at p<0.05. IG-F1=Insulin-like growth factor-1, CRL=Crown-ramp length, TP=Total protein

## Discussion

### Morphological characters of growth

Screening of the collected data about the BW and its ADC showed that early-weaned buffalo group suffered from a drop in weight gain post-weaning for 2 weeks after weaning. This finding was in agreement with Ali *et al*. [6] and Phillips *et al*. [11]. The authors concluded that poor growth rate in early 45-day-old weaned buffalo calves accompanied by a parallel depression in the rumen total volatile fatty acid concentration; the addition of mixture of acetic and propionic acids to stimuli rumen condition did not produce any improvement in growth. Decreased ADC in early-weaned buffalo calves post-weaning could be attributed down-regulation of IGF-1 production [12,13] due to poor post-weaning adaptation and incomplete rumenal development. On contrary, Pasha [14] stated that buffalo calves can be successfully weaned as early as 8 weeks of age without negatively affecting growth performance. The obtained results of height and ADC of height, girth, and ADC of girth were similar to that reported by Swali *et al*. [15] and Brickell *et al*. [16], respectively; however, screening the collected data of CRL and ADC of CRL (cm/day) showed lower values than that estimated by these authors.

### Clinical observations

Screening the collected data about weaning associated clinical signs suggested that ill-thriftiness was present in the early-weaned buffalo calves. Diarrhea was observed in only 2 out of 6 animals in early-weaned buffalo calves. Poor ruminal adaptation with subsequent nutrients deficiency is most likely cause of suboptimal growth in these calves. Our finding agreed with that reported by Abou El-Amaiem [17], who found that there were significant correlations between ill-thrift and young aged buffalo calves as well as diarrheic animals.

### Metabolic indices

#### Glucose

Glucose concentration is an indication of the energy status of individual animals. Reduced nutrient intake, periods of fasting and stress can cause reduction in blood glucose concentrations [18]. The highest blood glucose concentrations in both cow and late-weaned buffalo calves reflect the association between energy intake and growth in these groups. This finding is in accordance with previous studies in Holstein calves [19-21]. While late-weaned buffalo calves attained similar BW and blood glucose level to cow calves, the finding of the currect study may suggest that cow calves are superior to buffaloes in their growth rate. Furthermore, early-weaned buffalo calves had the lowest blood glucose level and growth rate and more likely to be culled, primarily due to ill-thriftiness. The positive link between blood glucose concentrations and time of weaning in buffalo calves may raise the question of how long farmers could keep buffalo calves with their dams, on the expense of their profitability from milk production.

#### Urea

In the present study, the blood urea level was positively correlated with CRL and girth while it showed weak and no positive association with height and weight, respectively. These results were in agreement with Swali *et al.*, [15]. Data showed temporary increase in blood urea level at weaning in early-weaned buffalo calves which could be attributed to the shortfall in energy during this times. This shortfall in energy could be met by the catabolism of body protein, which results in an increased blood urea concentration [22]. On the other hand, blood urea level showed significant increase in cow and late-weaned buffalo calves post-weaning compared with pre- and at-weaning times. It is known that urea may be elevated due to either a high protein intake from the initial diet or following catabolism of body protein reserves when energy intake is restricted [23].

#### Insulin

The obtained values of insulin level in the present study showed significant increase in all groups at pre-weaning time, however the highest insulin levels were present in late-weaned buffalo calves. By increasing the age of the calves, insulin levels decrease to normal. Higher insulin concentrations during pre-weaning time were associated with greater height and girth. A similar finding was reported by Swali *et al*. [15] which suggests that high insulin is associated with an alteration in body proportion.

#### IGF-1

IGF-1 regulates both skeletal and muscle development in growing cattle [24]. In the present study, IGF-1 concentration was positively associated with growth in all size parameters, reinforcing the important role of IGF-1 in the control of body growth. This is in accordance with previous findings that reported similar correlations between IGF-1 concentration at birth and subsequent growth in bull calves [25], lambs [22], and pigs [26]. We found that early-weaned buffalo calves had the lowest IGF-1 concentrations, and lowest body weight gain (ill-thrift) while cow and late-weaned buffalo calves had higher growth rate and higher levels of IGF-1. The positive association between growth and IGF-1 concentration reported in the current study is thought to reflect the association between energy intake and growth in these groups. IGF-1 is considered a suitable index of metabolic status as its concentration does not change in relation to time of feeding [27]. In milk-fed calves, insulin and glucose concentrations increase within 30 min, taking 4-6 h to return to basal concentrations post-prandially [27]. As it was not possible to be certain of the interval between feeding and blood sampling in this on-farm trial, IGF-1 concentration was considered as a reliable indicator for assessing the metabolic status of individual calves [16] and its association with growth.

#### Stress indices

Cortisol

Several studies assessed the level of cortisol in calves; however, there is wide variation in its value. In the present study, cortisol levels fall within the range reported by Aggarwal and Singh [18] and Hickey *et al*. [28]. The current study showed no significantly differences in cow calves group over the period of study suggesting that weaning is not a stressor in this animals or cortisol is not a reliable index for assessing stress. In contrast to that, the obtained data of cortisol levels showed slight increase at weaning in both groups of buffalo calves. These findings were in agreement with that reported by Kim *et al*. [19] suggesting that buffalo calves may be more vulnerable to stress than cow calves because they are highly social animals with strong instincts and closely bonded with their dams [20]. Although buffalo calves may subject to stress of weaning, cortisol level showed no association with growth parameters in the present study.

Protein profile

Collected data about protein profile were similar to that reported by El-Bahr and El-Deeb [20]. The significant increase of the level of TP, albumin and globulin in late-weaned buffalo calves group strongly suggests that these animals are well adapted to weaning compared with the other two groups. Increase in TP values with increase in age has been reported by other workers [11]. Data showed no significant difference in the A/G ratio among the studied groups during preweaning time while it significantly decrease in buffalo groups compared with cow calves postweaning.

WBCs picture

Total leukocytic count, neutrophil %, lymphocyte %, and N/L ratio showed no significant changes among groups in the present study. Similar finding was reported by Earley *et al*. [29]. Within the same group, early-weaned buffalo calves showed significant increase in N/L ratio at pre-weaning and at-weaning periods. This finding was in agreement with Kim *et al*. [19]. The authors suggested that weaning affects leukocyte level and that the N/L ratio may be effective biomarker of stress responses. Lynch *et al*. [30] suggested that the glucocorticoids are a main contributing factor to the alteration of N/L ratio. Therefore, changes in the N/L ratio are thought to be a potential biological indicator of stress and disease susceptibility.

## Conclusion

Suboptimal growth rate (ill-thrift) in early-weaned buffalo calves is attributed to low blood levels of growth metabolites, in particularly, IGF-1. In addition, the strong positive associations between concentrations of IGF-1 and morphological characters of growth suggest that IGF-1 is a reliable test for assessing metabolic status of individual calves. This may be diagnostically useful as it is often not possible to obtain accurate information on nutrient intakes or disease at a farm level.

## Authors’ Contributions

NMA and AES have formulated the research plan. HZH conducted clinical examination and collected samples. NMA analyzed samples. AES and HZH drafted the manuscript and NMA revised and submitted it. All authors read and approved the final manuscript.
